# Complete remission of recurrent human papillomavirus-associated cervical cancer managed with camrelizumab and nanoparticle albumin-bound paclitaxel as second-line treatment: a case report

**DOI:** 10.3389/fimmu.2025.1679950

**Published:** 2025-12-02

**Authors:** Liping Sun, Juan Lang, Xueya Wu, Dan Shi, Jiefeng Sang, Zhongkui Xiong

**Affiliations:** 1 Department of Pathology, Shaoxing People’s Hospital, Shaoxing, Zhejiang, China; 2 School of Medicine, Shaoxing University, Shaoxing, Zhejiang, China; 3 Department of Radiation Oncology, Shaoxing Second Hospital, Shaoxing, Zhejiang, China; 4 Department of Pathology, Shaoxing Second Hospital, Shaoxing, Zhejiang, China; 5 Department of Radiology, Ganyu District People’s Hospital of Lianyungang City, Lianyungang, Jiangsu, China

**Keywords:** cervical cancer, human papillomavirus, second-line therapy, camrelizumab, nanoparticle albumin-bound paclitaxel

## Abstract

Cervical cancer is the fourth most prevalent oncological condition affecting the global female population in 2022, considering both disease occurrence and fatality rates. Although surgical intervention is the curative approach for early stage cervical cancer, recurrent progression is associated with unfavorable clinical outcomes. The current therapeutic protocols outlined in the National Comprehensive Cancer Network Guidelines 4.2025 edition propose that for second-line or subsequent therapies of cervical carcinoma, prioritized protocols incorporate pembrolizumab administration specifically for patients demonstrating high tumor mutational burden characteristics, positive for programmed cell death ligand 1 expression, or exhibiting microsatellite instability-high/mismatch repair deficiency molecular profiles. Other proposed therapeutic approaches include bevacizumab, paclitaxel, and nanoparticle albumin-bound paclitaxel (nab-paclitaxel). To date, no standardized systemic combination protocol has been established for the management of recurrent/metastatic cervical carcinoma after first-line treatment. The clinical application of camrelizumab combined with nab-paclitaxel as a second-line intervention for recurrent human papillomavirus (HPV)-associated cervical cancer remains rare in existing medical literature. This case report documents complete remission achieved through second-line camrelizumab combined with nab-paclitaxel therapy in a 65-year-old Chinese female with recurrent HPV-associated cervical cancer with positive programmed cell death ligand 1 (PD-L1) in whom initial treatment failed. Clinical outcomes included the disease-free survival of 22 months, accompanied by the first progression-free survival (PFS1) of 10 months and the PFS2 of 58 months. The overall survival was recorded at 92 months. The patient continues to undergo active clinical surveillance. Our case report illustrates that second-line immunochemotherapy utilizing camrelizumab in combination with nab-paclitaxel exhibits notable efficacy and manageable safety profile.

## 1 Introduction

Cervical cancer is the fourth most prevalent malignancy affecting women globally, considering both occurrence and mortality rates. Recent statistics from 2022 reveal that this disease will account for approximately 660,000 diagnoses and 350,000 fatalities worldwide (1). Current epidemiological projections indicate that these numbers will increase to 760,082 diagnoses (14.8% increase) and 411,035 deaths (17.8% increase) by 2030 (2). Patients diagnosed with advanced or metastatic cervical cancer have a five-year overall survival (OS) rate of <5% (3). Chronic infection with high-risk human papillomavirus (HPV) is the principal causative factor of cervical carcinogenesis, initiating immune escape pathways that accelerate neoplastic development and progression (4). Oncogenes E6 and E7 encoded by HPV stimulate topoisomerase I overexpression, subsequently triggering the cyclic GMP–AMP synthase–programmed cell death ligand 1 (PD-L1) signaling axis during malignant progression (5). HPV16 E6 and E7 oncoproteins can facilitate immune evasion mechanisms in cervical carcinoma through interactions with the miR-142-5p/PD-L1 signaling pathway (6). HPV-associated cervical malignancies demonstrate superior clinical responsiveness to immune checkpoint blockade regimens compared to HPV-independent cervical cancers (7).

Although surgical intervention is effective in treating early-stage cervical cancer, individuals with recurrence often encounter unfavorable clinical outcomes and have limited therapeutic options (8). Several patients with locally advanced cervical cancer (LACC) develop localized recurrence or metastasis after radiation therapy (9). The combination of cisplatin and paclitaxel administered during concurrent chemoradiotherapy (CCRT) failed to significantly enhance survival outcomes in patients with LACC. Nevertheless, the hematotoxic effects linked to combined chemotherapy regimens tend to be more severe, while remaining clinically controllable. Therefore, cisplatin monotherapy is the preferred treatment for CCRT (10). The integration of platinum-based cytotoxic agents with radiotherapy results in superior survival outcomes in cervical cancer (11). A multicenter EMBRACE-I cohort analysis systematically evaluated metastatic patterns following chemoradiation therapy combined with magnetic resonance imaging (MRI)-guided adaptive brachytherapy for LACC. The cumulative rate of distant metastasis was 14%, with pulmonary metastases emerging as the predominant site (26%), followed by mediastinal lymph node involvement (15%) and osseous spread (10%). Significant predictors include non-squamous tumor histopathology, presence of nodal metastases at initial diagnosis, and extensive brachytherapy treatment volumes (12). In patients diagnosed with early-stage cervical carcinoma who undergo radical hysterectomy combined with pelvic lymph node dissection, the incidence of isolated para-aortic lymph node involvement is relatively uncommon when the pelvic lymph nodes are positive, whereas the common iliac nodes remain negative, particularly after adjuvant pelvic chemoradiotherapy (13).

Multiple clinical parameters have demonstrated potential associations with PD-L1 positivity, including HPV-positive status, squamous cell histopathology, later disease stages, larger tumor dimensions, lower histological differentiation grades, presence of metastatic lesions, multiparous obstetric history, prior termination procedures, and previous exposure to chemotherapeutic agents (3). Elevated PD-L1 levels in patients with cervical carcinoma are correlated with less favorable clinical outcomes (3). The KEYNOTE-826 clinical investigation revealed a marked improvement in both progression-free survival (PFS) and OS outcomes when pembrolizumab was administered compared to a placebo in individuals diagnosed with persistent, recurrent, or metastatic cervical carcinoma undergoing chemotherapy regimens with or without bevacizumab administration (14).

These findings align with the recommendations outlined in the fourth edition of the National Comprehensive Cancer Network (NCCN) 2025 Clinical Practice Guidelines. For patients with recurrent or metastatic (R/M) cervical cancer (squamous cell carcinoma, adenocarcinoma, or adenosquamous subtypes) with a PD-L1-positive status, current guidelines suggest prioritized treatment approaches for initial therapy as follows: 1) pembrolizumab combined with cisplatin/carboplatin and paclitaxel, optionally combined with bevacizumab; 2) cisplatin/carboplatin administered alongside paclitaxel and bevacizumab; and 3) atezolizumab in conjunction with cisplatin/carboplatin, paclitaxel, and bevacizumab. These combination regimens integrate immunotherapy agents with conventional chemotherapy and anti-angiogenic therapies to optimize therapeutic outcomes.

Currently, no established systemic combined treatment exists for R/M cervical cancer following failure of first-line therapy (15). The NCCN Guidelines Version 4.2025 advocate pembrolizumab (specifically for tumor mutational burden high, PD-L1-positive, or microsatellite instability-high (MSI-H)/mismatch repair deficiency tumor profiles) as the principal option for second-line or subsequent therapies. These recommendations are complemented by alternative protocols incorporating bevacizumab, paclitaxel, and nanoparticle albumin-bound paclitaxel (nab-paclitaxel) along with other chemotherapeutic agents. Camrelizumab, a human-engineered programmed cell death (PD-1) inhibitor targeting distinct epitopes from nivolumab and pembrolizumab, has demonstrated therapeutic potential in cervical cancer (16). A retrospective analysis of combination therapy with camrelizumab, nab-paclitaxel, and apatinib showed a partial response (PR) in 26.7% of the participants and stable disease (SD) in 20.0%. The overall response rate was 26.7%, and the disease control rate (DCR) of 46.7%. Survival outcomes indicated a median PFS and OS of 3.0 and 8.0 months, respectively. Treatment was not discontinued because of intolerable toxicity (17). A randomized Phase II study demonstrated that the median PFS was significantly prolonged with the combination of camrelizumab and famitinib, a multitarget receptor tyrosine kinase inhibitor, in comparison to camrelizumab alone or chemotherapy in R/M cervical cancer (8.1 months vs. 4.1 months and 2.9 months) (18).

To date, few documented cases have been reported of patients with recurrent HPV-associated cervical carcinoma receiving second-line camrelizumab combined with nab-paclitaxel therapy. In July 2020, a 65-year-old Chinese woman presented with epigastric discomfort and episodes of nausea and emesis at Shaoxing Second Hospital. Clinical evaluation revealed an afebrile status with marked fatigue, appetite, absence of productive cough or sputum production, no thoracic discomfort, and mild exertional dyspnea. No overtly positive findings were identified during the physical examination. Laboratory tests conducted upon admission revealed anemia, leukocytosis, renal impairment, urinary tract infection, and elevated tumor marker levels. This case study details a patient with recurrent HPV-associated cervical cancer with positive PD-L1 who achieved complete remission (CR) with camrelizumab and nab-paclitaxel after initial treatment failed.

## 2 Case report

**Figure 1:**
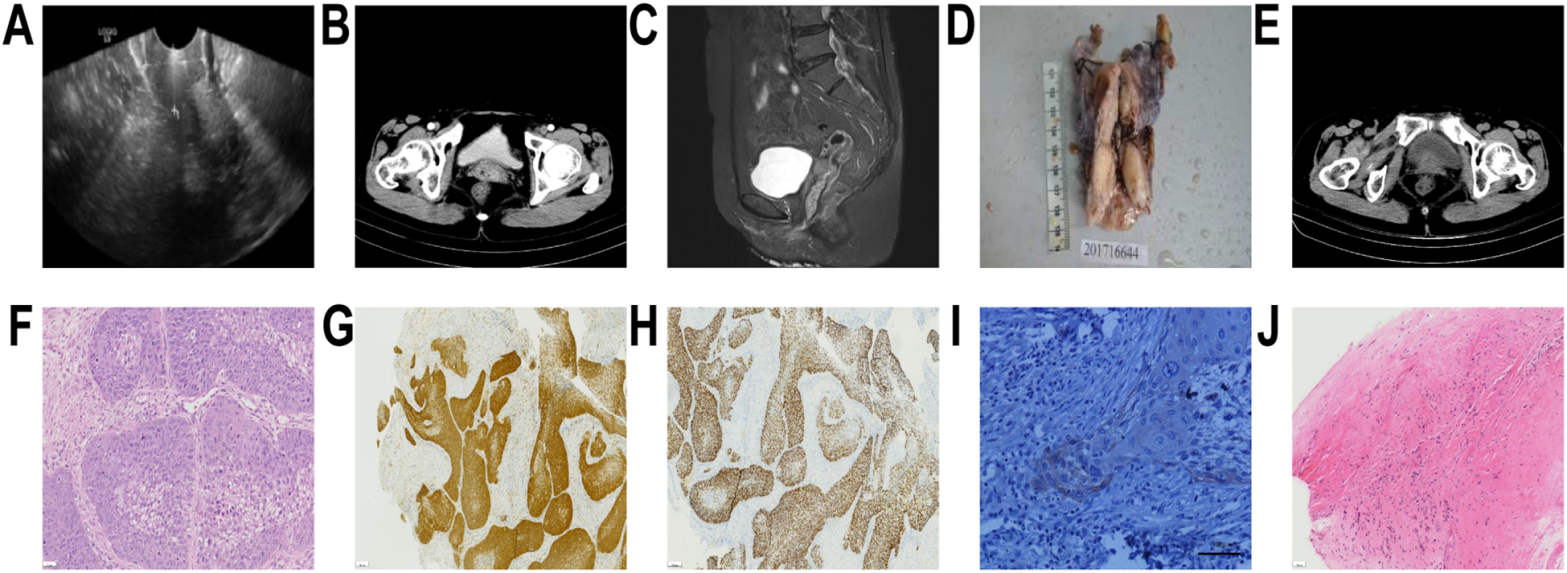
Imaging and pathological results. **(A–C)** Imaging results. **(A)** An ultrasound revealed a well-defined hypoechoic lesion in the cervical region, measuring 29 × 21 mm on September 4, 2017. **(B)** Computed tomography scan conducted on September 7, 2017 revealed an enlarged cervix with heterogeneous enhancement and a thickened vaginal wall. **(C)** On September 7, 2017, magnetic resonance imaging (MRI) revealed an enlarged cervix with mixed signal and a diameter of approximately 27 mm. **(D)** Image of postoperative gross specimen on September 13, 2017. **(E)** No enlargement of the vaginal stump was observed as reported by abdominal CT on May 18, 2020. **(F–J)** Pathological results. **(F)** Postoperative hematoxylin and eosin staining on July 17, 2019. **(G)** P-16 on July 17, 2019. **(H)** P-40 on July 17, 2019. **(I)** PD-L1 CPS 12 on July 17, 2019. **(J)** Vaginal wall biopsy conducted on May 15, 2020.

On September 4, 2017, an ultrasound scan identified a distinct hypoechoic lesion in the cervical area, measuring 29 × 21 mm (**Figure 1A**), displaying enhanced vascular patterns with marked blood flow activity. Diagnostic testing performed on the same day showed that cervical secretions demonstrated a positive reaction (++) for HPV-16 by molecular analysis. Histopathological analysis on September 6, 2017 confirmed the squamous epithelium of the cervical canal with focal carcinomatous transformation (**Table 1**). Imaging studies performed the following day showed cervical abnormalities, and computed tomography (CT) revealed cervical enlargement with heterogeneous contrast enhancement and vaginal wall thickening (**Figure 1B**). Concurrent MRI revealed an expanded cervical structure measuring approximately 27 mm in diameter with a heterogeneous signal (**Figure 1C**). Post-contrast imaging showed pronounced contrast uptake in the affected area, with the vaginal walls in the mid-to-upper segments displaying notable thickening and substantial enhancement. Pelvic lymph node assessment revealed no pathologically enlarged lymph nodes. The clinical diagnosis of cervical carcinoma was confirmed on September 12, 2017, prompting surgical intervention at Shaoxing Second Hospital, involving radical hysterectomy with bilateral salpingo-oophorectomy and comprehensive pelvic lymphadenectomy. Histopathological evaluation conducted on September 13, 2017 demonstrated invasive squamous cell carcinoma within the cervical tissue (moderately poorly differentiated) in the surgical specimens (**Table 1**). Histopathological evaluation confirmed stage IIa cervical cancer according to the revised 2018 International Federation of Gynecology and Obstetrics (FIGO) staging system. Postoperative management included radiation therapy administered through 25 fractional doses, totaling 45 Gy delivered via 6 MV X-ray technology. Despite completing five radiotherapy sessions, the treatment course was discontinued because of physical limitations. The patient underwent radical surgical resection of the right breast malignancy in 2015 and subsequently received three cycles of adjuvant chemotherapy. Presently, the patient maintains a therapeutic regimen of letrozole (2.5 mg) daily as part of ongoing endocrine treatment. On July 17, 2019, a vaginal stump biopsy performed during gynecological evaluation at Shaoxing Second Hospital revealed histopathological evidence of squamous cell carcinoma (**Table 1**). These diagnostic findings were subsequently confirmed during follow-up clinical assessments conducted 2 days later on July 19, 2019. On July 20, 2019, MRI revealed a nodular vaginal lesion measuring approximately 17 × 22 mm that demonstrated marked enhancement after contrast injection. This finding led to a diagnosis of cervical cancer recurrence, staged as stage IIa according to the FIGO 2018 criteria. Radiotherapy commenced on July 24, 2019, with the patient receiving fractionated 6MV X-ray irradiation, totaling DT = 45 Gy/25 Fx, achieving treatment completion in August 2019. The patient received an intravenous chemotherapy regimen using cisplatin at a dose of 40 mg, with treatment sessions recorded on July 8 and August 14, 2019. Subsequently, a high-dose-rate intracavitary brachytherapy protocol was implemented from September 2–15, 2019, delivering a total dose of 25 Gy through five fractional treatments.

**Table 1:** Pathological results.

Time	Pathological specimen	Pathological results
September 6, 2017	Cervical puncture specimen	Marked dysplastic changes in the cervical canal’s squamous epithelium with focal carcinomatous transformation.
September 13, 2017	Post-surgical tumor specimen	Image of postoperative gross specimen was presented (**Figure 1D**), Histopathological evaluation demonstrated invasive squamous cell carcinoma within the cervical tissue (moderately–poorly differentiated) from surgical specimens including the entire uterus, bilateral adnexa, and dissected pelvic lymph nodes. The neoplasm measured 2.5 × 1.6 cm in maximum dimensions penetrating deeply into the cervical fibromuscular wall (exceeding two-thirds thickness) with a maximal invasive depth of 1.4 cm. Cervical canal involvement was observed histologically, although comprehensive examination revealed absence of conclusive evidence regarding neurovascular invasion or lymphovascular space infiltration. The uterine cavity showed no evidence of malignancy. Several leiomyomas were noted in both subendometrial and intramural locations, measuring between 0.2 and 1.2 cm. Endometrial tissue displayed features consistent with the proliferative phase, whereas both the bilateral parametrial regions and adnexal structures showed no signs of neoplastic infiltration. Pathological examination of regional lymph nodes demonstrated no evidence of metastasis across the examined anatomical sites as follows: right common iliac nodal group (0/2 positive), right deep inguinal nodal basin (0/4), right internal obturator chain (0/4), left common iliac node (0/1), left external iliac cluster (0/2), left deep inguinal node collection (0/3), and left internal obturator group (0/6). Histopathological analysis of the right external iliac specimen identified exclusively fibroadipose tissue components. Immunohistochemical analysis (case ID: 20170882) revealed positivity for C5/6, P40, CK (H), CEA, P16, E-Cad, and P53 markers, with focal expression of CyclinD1 and a high Ki-67 proliferation index of 75%. Negative results were observed for CK8 and CK (L) markers.
July 17, 2019	Vaginal stump biopsy	Histopathological evidence of squamous cell carcinoma (**Figure 1F**). Subsequent immunohistochemical analysis demonstrated positive staining for CK5/6, CK8, CEA, P16 (**Figure 1G**), and E-cadherin markers, while showing negative reactivity for P40 (**Figure 1H**), CK(L), and P53. PD-L1 CPS 12 (**Figure 1I**). The proliferation index assessed by Ki-67 immunostaining reached approximately 80% positivity.
May 15, 2020	Vaginal stump biopsy	Histopathological analysis demonstrated focal fibrous tissue regions containing dispersed lymphocyte and plasma cell infiltrations, with adjacent squamous epithelial zones being concurrently identified (**Figure 1J**).

**Figure 2:**
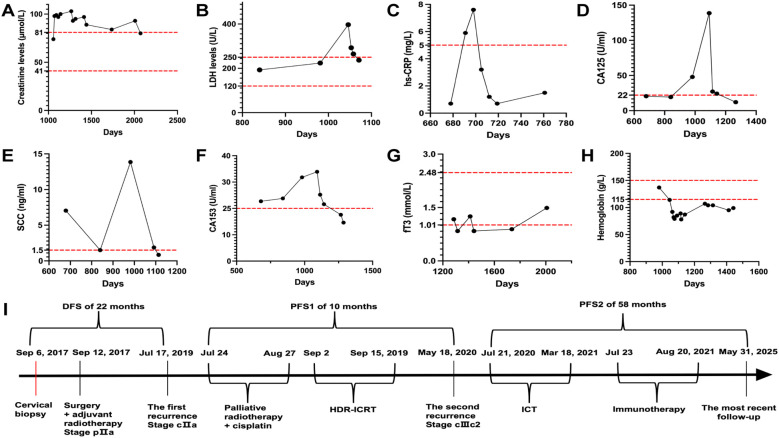
Course timeline and laboratory test results of the patient. **(A)** Creatinine levels: Following the initiation of immunochemotherapy, the patient’s serum creatinine concentrations persistently exceeded normal reference values between August 12, 2020 and March 7, 2023. **(B)** Concurrently, longitudinal monitoring revealed a gradual elevation in lactate dehydrogenase (LDH) activity from July 18, 2020 to July 30, 2023. Post-administration of two treatment cycles, biochemical parameters demonstrated marked improvement with LDH values normalizing to reference ranges by August 12, 2020. **(C)** Between July 29, 2020, and August 5, 2023, test findings revealed a marked elevation in high-sensitivity C-reactive protein levels. After anti-infective therapy, concentrations normalized by August 12, 2020. **(D)** Elevated CA125 concentrations were first detected on May 15, 2020, peaking at 138.7 U/mL by September 2, 2020. Therapeutic intervention subsequently reduced these levels, with complete normalization achieved by February 23, 2021. **(E)** Concurrently, squamous cell carcinoma antigen concentrations initiated an upward trajectory on July 17, 2019, attaining peak measurements of 13.85 ng/mL by May 14, 2020. Subsequent medical management facilitated antigen level reduction, with values returning to baseline levels by September 24, 2020. **(F)** CA153 concentrations gradually increased starting from July 17, 2019 and reached its highest recorded level of 33.9 U/mL by September 2, 2020, and thereafter progressively decreased through therapeutic intervention, stabilizing within standard parameters by February 23, 2021. **(G)** Initial laboratory assessments on April 13, 2020 revealed reduced triiodothyronine concentrations. Following therapeutic intervention with euthyroxine supplementation, follow-up evaluations conducted on July 20, 2021 showed normalization of thyroid hormone parameters (**Figure 2**). **(H)** Prior to initiating immunochemotherapy on July 18, 2020, hematological monitoring recorded a hemoglobin value of 114 g/L. Following the first cycle of immunochemotherapy, the patient’s hemoglobin concentrations persistently remained below the standard reference range for >12 months (August 4, 2020 to August 18, 2021). **(I)** Course timeline illustrating the treatments administered. Clinical management strategies, therapeutic response assessments, and treatment-related complications are comprehensively illustrated.

**Figure 3:**
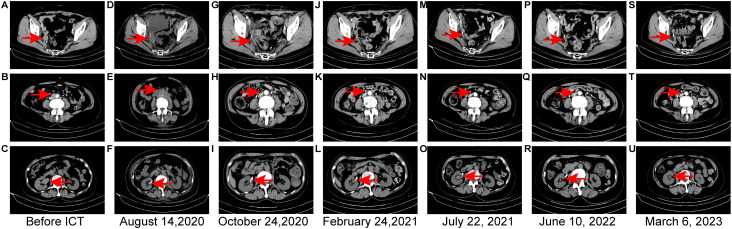
Computed tomography (CT) images during second-line therapy. **(A–C)** CT images before second-line therapy. **(A)** Enlarged lymph node adjacent to the right pelvic wall, significant enhancement following contrast administration performed on May 18, 2020. **(B)** Enlarged lymph node in the retroperitoneal area, significant enhancement following contrast administration performed on May 18, 2020. **(C)** Distended right renal pelvis and ureter on July 18, 2020. **(D–F)** CT on August 14, 2020 after two cycles of immunochemotherapy. **(D)** Enlarged lymph node adjacent to the right pelvic wall. **(E)** Enlarged lymph node in the retroperitoneal area. **(F)** No evidence of dilatation in the right renal pelvis or ureter following the insertion of the J-tube. **(G–I)** CT images on October 24, 2020 after completing four cycles of immunochemotherapy. **(G)** Metastatic lymph nodes adjacent to the right pelvic wall significantly decreased in size. **(H)** Metastatic lymph nodes in the retroperitoneal region significantly decreased in size. **(I)** No evidence of dilatation in the right renal pelvis or ureter. **(J–L)** CT images on February 24, 2021 after completing five cycles of immunochemotherapy. **(M)** Metastatic lymph nodes adjacent to the right pelvic wall significantly decreased in size. **(N)** Metastatic lymph nodes in the retroperitoneal region significantly decreased in size. **(O)** No evidence of dilatation in the right renal pelvis or ureter. **(M–O)** CT images on July 22, 2021 after completing six cycles of immunochemotherapy showed that the metastatic lymph nodes in the retroperitoneal region and adjacent to the right pelvic wall had reduced in size relative to prior imaging. Furthermore, medial displacement of the right ureter was noted. **(P–R)** CT scan conducted on June 10, 2022–2 years following the diagnosis of the second recurrence showed that multiple small lymph nodes in the retroperitoneal region and adjacent to the right pelvic wall. **(S–U)** CT scan conducted on March 6, 2023.

In July 2020, a 65-year-old Chinese woman patient presented with epigastric discomfort and episodes of nausea and emesis at Shaoxing Second Hospital. Clinical evaluation revealed an afebrile status with marked fatigue and appetite, absence of productive cough or sputum production, no thoracic discomfort, and mild exertional dyspnea. No overtly positive findings were identified during the physical examination. Laboratory test results upon admission are presented in **Figure 2**. Analgesic therapy consisting of oral oxycodone 40 mg administered every 12 hours effectively maintained abdominal pain control, with a numerical rating scale score of 2. Following the initial diagnosis, the patient remained alert while experiencing cognitive exhaustion, accompanied by disrupted sleep patterns and preserved urinary and defecatory functions, with a documented reduction in body weight of 5 kg. Histopathological analysis of a vaginal wall specimen obtained on May 15, 2020 demonstrated focal fibrous tissue regions containing dispersed lymphocytes and plasma cell infiltrations, with adjacent squamous epithelial zones being concurrently identified (**Table 1**). Multiple enlarged lymph nodes, measuring up to 21 mm, were observed near the right pelvic wall (**Figure 3A**) and within the retroperitoneal space (**Figure 3B**). These structures exhibited marked contrast enhancement during a contrast-enhanced CT scan performed on May 18, 2020 (**Figure 1E**). Subsequent imaging studies from July 18, 2020, revealed dilatation affecting both the right renal pelvis and the ureter (**Figure 3C**). At this juncture, the patient received a clinical diagnosis indicating a second instance of cervical cancer recurrence, with disease progression classified as stage IIIc2 according to the FIGO 2018 staging criteria. Urinalysis conducted on July 18, 2020 was positive for *Streptococcus agalactiae*. The therapeutic regimen included daily intravenous administration of 0.15 gentamicin, which was maintained from July 18–30, 2020. Between July 21, 2020 and March 18, 2021, the patient received six treatment cycles combining immunotherapy and chemotherapy, with each 3-week cycle consisting of 200 mg camrelizumab and 300 mg nab-paclitaxel. Therapeutic interventions were delivered on the following dates: July 21, August 6, September 4, September 25, and October 22 (2020) and March 18, 2021. Following this regimen, the patient received two additional cycles of monotherapy with camrelizumab at 200 mg doses administered triweekly, specifically on July 23 and August 20, 2021. The therapeutic outcomes demonstrated SD as assessed by CT imaging (**Figures 3D–F**) on August 14, 2020, based on the immune-related Response Evaluation Criteria in Solid Tumors criteria after two cycles of immunochemotherapy. The therapeutic outcome was classified as a PR following the administration of four cycles of immunochemotherapy, as determined by CT imaging conducted on October 24, 2020 (**Figures 3G–I**). Following five cycles of immunochemotherapy, the therapeutic outcome was evaluated as a PR based on CT imaging conducted on February 24, 2021 (**Figures 3J–L**). After six cycles of combined immunotherapy and chemotherapy, a subsequent CT imaging assessment conducted on July 22, 2021 (**Figures 3M–O**) indicated a therapeutic response that confirmed CR. At 24 months after the diagnosis of the second recurrence, imaging studies conducted on June 10, 2022 (**Figures 3P–R**) revealed a therapeutic response that confirmed CR. The therapeutic outcome evaluation confirmed CR, with subsequent surveillance imaging demonstrating the persistent stability of these findings (**Figures 3S–U**). Changes in laboratory test results during the second-line therapy phase are illustrated in **Figure 2**, and treatment-associated adverse effects were documented according to the Common Terminology Criteria for Adverse Events v4.03: grade 2 anemia (**Figure 2**) and grade 2 hypothyroidism (**Figure 2**).

In summary, for the case of R/M cervical cancer treated with a second-line therapy involving camrelizumab in combination with nab-paclitaxel, the disease-free survival (DFS) duration of 22 months was observed. The PFS1 lasted for 10 months, while the PFS2 extended to 58 months. The OS was recorded at 92 months.

## 3 Discussion

A thorough analysis was conducted to examine the likelihood of recurrence following surgical intervention and the long-term outcomes in individuals diagnosed with early stage cervical carcinoma. Pathological evaluation revealed no evidence of high-risk features, including margin positivity, parametrial involvement, or metastatic lymph node involvement. This study specifically targeted the identification and assessment of intermediate prognostic indicators. First, regarding tumor dimensions, for patients with early stage cervical carcinoma lacking high-risk features who undergo radical surgery without adjuvant therapy, the likelihood of disease recurrence generally remains relatively limited. However, specific pathological characteristics, including lesions > 2 cm in diameter or poorly differentiated tumors (grade 3), exhibit elevated recurrence probabilities according to clinical evidence (19). Second, the extent of stromal infiltration within the cervical tissue has been established as a critical prognostic parameter for cervical cancer outcomes, as demonstrated in recent oncological research (20). The administration of supplementary carboplatin-paclitaxel chemotherapy following conventional cisplatin-containing chemoradiation in patients with non-selected advanced-stage cervical carcinoma was associated with heightened immediate toxicities and demonstrated no significant enhancement in long-term survival outcomes (21). In patients diagnosed with stage IB2 cervical cancer under FIGO 2018 criteria who did not undergo adjuvant treatment, studies have identified deep stromal invasion within the cervix as a significant predictor of disease recurrence (22). Regarding tumor characteristics, histological analysis revealed that squamous cell carcinoma (SCC) cases generally demonstrate improved clinical outcomes compared to non-squamous carcinoma variants. This prognostic advantage may stem from typically slower progression patterns and enhanced responsiveness of SCC to conventional therapeutic approaches compared to other histological subtypes. Among patients diagnosed with SCC, both initial SCC antigen levels observed during diagnosis and the occurrence of lymph node metastases have been identified as independent determinants of the probability of developing distant metastases (23).

The mechanism by which postoperative adjuvant chemotherapy influences recurrence rates and long-term outcomes in patients with cervical cancer exhibiting intermediate-risk factors (IRFs) remains unclear. Current research indicates that among patients with stage IA2–IIA cervical carcinoma undergoing radical hysterectomy with pelvic lymphadenectomy, those pathologically classified as having an intermediate-risk postoperatively demonstrate varying susceptibility. Specifically, individuals diagnosed with SCC exhibiting all three IRFs and non-SCC patients demonstrating two or more IRFs showed elevated recurrence probabilities, suggesting distinct therapeutic considerations for these subgroups within the intermediate-risk category. Adjuvant chemotherapy is a viable therapeutic approach for managing the recurrence risk in this patient cohort (24). For women diagnosed with stage IB1–IIA2 cervical SCC (per FIGO 2009 staging) who underwent radical hysterectomy with pelvic lymphadenectomy and showed no pathological involvement in lymph nodes, resection margins, or parametrial tissues, observational management without adjuvant therapy could yield comparable survival outcomes when IRFs (as outlined in the GOG-92 trial’s Sedlis criteria) are present (25). For patients with early-stage cervical cancer exhibiting IRFs, as defined by the Sedlis criteria, comparative analyses revealed that the likelihood of disease recurrence and OS outcomes demonstrated comparable patterns among those receiving postoperative interventions versus those managed through an observation-only approach (26).

At 22 months after the initial therapeutic intervention, tumor recurrence was observed in the patient’s vaginal stump. Analysis of cervical cancer cases with post-radiation recurrence (primary or adjuvant) revealed distinct recurrence patterns: DM predominated (59.5%), with combined-site recurrence occurring in 21.5% of cases. Central recurrences involving the cervical or vaginal stump regions accounted for 10.7%, whereas pelvic recurrences affecting the lymph nodes or lateral walls comprised 8.3% of the cases (27). Regarding local therapy, individuals presenting with oligometastatic recurrence should be comprehensively assessed to investigate therapeutic interventions with curative potential (28). Tumor dimensions, categorized as large (≥3 cm), medium (<3 cm), or small (non-palpable), have emerged as crucial prognostic indicators of vaginal stump recurrence in cervical carcinoma. Current guidelines suggest that patients with nonpalpable lesions undergo exclusive brachytherapy (29). High-dose-rate brachytherapy has demonstrated efficacy as a therapeutic approach for cervical cancer recurrence in the vaginal stump regions, achieving 3-year OS rates of 100% along with local control and PFS rates of 82.8% and 76.8%, respectively (30). For patients with recurrent or regionally persistent cervical malignancies, integration of surgical intervention with intraoperative radiotherapy emerged as a viable strategy for appropriately selected cases (31). Patients with recurrent locoregional cervical cancer undergoing IMRT-based salvage therapy have demonstrated favorable clinical outcomes (32). Recurrence location and systemic inflammatory response index have been identified as key determinants of survival outcomes. Targeted regional radiotherapy is particularly effective in managing localized recurrences (32). Combined-modality therapy utilizing 3D image-guided brachytherapy with external beam radiation achieves optimal tumor management with manageable side effects in recurrent gynecological malignancies (33). Regarding systemic therapy, platinum-based chemotherapy serves as the cornerstone of therapeutic intervention for recurrent cervical carcinoma (34). The standard therapeutic protocol for R/M cervical cancer typically involves a regimen combining cisplatin, paclitaxel, and bevacizumab; however, this treatment strategy yields modest clinical outcomes, highlighting substantial unmet needs in oncological management (35). Clinical investigations analyzing palliative chemotherapy outcomes in patients with recurrent cervical cancer have revealed a median PFS of 8.4 months and an OS of 10.3 months in real-world settings (36).

In 1976, Pattillo et al. published a report detailing the application of immunotherapy and chemotherapy in the management of recurrent cervical cancer (37). Clinical trials have demonstrated that pembrolizumab plus chemotherapy regimens exhibit prolonged PFS and OS with acceptable safety profiles compared to placebo-controlled groups, particularly in patient cohorts stratified by bevacizumab treatment history (38). The integration of pembrolizumab into chemotherapy protocols, either administered alongside bevacizumab or as monotherapy, has demonstrated significant survival benefits across various patient subsets with persistent or R/M cervical malignancies (39).

Seven months after completion of afterloading radiotherapy, the patient developed secondary disease progression manifested by metastatic involvement of the pelvic and para-aortic lymph nodes alongside tumor-induced hydronephrosis. Importantly, no tumor recurrence was detected at the vaginal stump. Previous studies have examined the occurrence of hydronephrosis after chemoradiotherapy in patients with LACC. Initial evaluations revealed pre-existing hydronephrosis in 10.9% of the renal units, with 38% showing resolution post-treatment. Radiotherapy is associated with an 8% incidence rate of hydronephrosis. Subsequent monitoring identified newly developed hydronephrosis in 15.8% of renal units, with tumor recurrence twice as prevalent as radiation-induced cases (2:1 proportion) based on clinical findings (40).

In cases of R/M cervical cancer in which progression occurs following first-line treatment, second-line therapeutic strategies require careful consideration. Until recently, the application of non-cytotoxic systemic therapy has yielded only limited success (41). In chemotherapy, the duration of platinum-free intervals exceeding 24 months is a critical threshold for clinical decision making. This parameter not only guides medication selection across various gynecological cancers, but also establishes the differentiation criteria between platinum-sensitive and platinum-resistant cervical carcinoma subtypes (42). The extended treatment-free period following platinum-based regimens provides a valuable framework for determining appropriate therapeutic options in subsequent treatment phases. In platinum-sensitive patients, a Gynecologic Oncology Group phase II clinical trial investigated pemetrexed as secondary therapy for persistent/recurrent cervical carcinoma, demonstrating a median PFS of 3.1 months and OS reaching 7.4 months (43). For advanced/recurrent cervical cancer management, cisplatin-topotecan combination therapy as a secondary treatment achieved an extended median PFS (4.6 months) with a median OS duration of 14.1 months (44). Clinical trials evaluating weekly administration of topotecan as a secondary or tertiary therapeutic approach for R/M cervical carcinoma have demonstrated no objective responses (complete or partial). SD was observed in 27.7% of the cases, with patients showing a median PFS of 3.5 months and an OS of 7 months (45).

Regarding targeted therapy, in a phase II clinical trial investigating apatinib, an innovative angiogenesis inhibitor targeting vascular endothelial growth factor receptor-2, as a secondary treatment for R/M cervical carcinoma, researchers observed a median PFS of 5.13 months and an OS of 12.3 months (46). A preliminary clinical trial evaluated the combination of nimotuzumab with monochemotherapy as a second-line therapeutic approach in patients with persistent, or R/M cervical carcinoma. The investigation reported median PFS and OS of 163 and 299 days, respectively (47). Regarding immunotherapy with HPV vaccine, during chemoradiotherapy, HPV cfDNA concentrations demonstrate dynamic variations. Post-treatment measurements of HPV cfDNA have been established as reliable indicators of predicting recurrence-free survival outcomes (48). Declining HPV ctDNA concentrations are significantly associated with therapeutic responsiveness in R/M cervical cancer (49). Cervical cancer is predominantly associated with HPV genotypes 16 and 18, which have been identified as major causative agents of cervical carcinogenesis (50). In a phase I/IIa clinical investigation evaluating BVAC-C therapy for patients with HPV-16/18-positive cervical cancer who showed disease progression following initial platinum-based chemotherapy regimens, researchers observed a median PFS of 5.8 months and an OS of 17.7 months (51). Subsequent research involving MEDI0457 combined with durvalumab for R/M HPV-associated neoplasms showed favorable safety characteristics and manageable tolerability in patients with advanced HPV-16/18-related malignancies during phase II testing. However, despite demonstrating a clinically meaningful DCR, the cervical cancer trial was ultimately halted because of suboptimal therapeutic responses among participants (52). A retrospective analysis of 40 patients who received primary concurrent chemoradiotherapy for endocervical adenocarcinomas (stages I–IVA) revealed distinct biological behaviors. Through comprehensive histopathological evaluation, the tumors were classified as either HPV-associated or HPV-independent cervical cancer, with HPV-independent cases exhibiting markedly elevated parametrial involvement (94.4% vs. 45.5%, p=0.001). CR was achieved in 57.5% of participants, with a notably higher occurrence in HPV-associated cases (81.8% vs. 27.8%, p=0.001). The HPV-independent cohort demonstrated elevated recurrence frequency (88.9% vs. 50.0%, p=0.016) alongside diminished three-year PFS (16.7% vs. 49.8%, p=0.001), reduced distant metastasis-free survival (38.1% vs. 80.8%, p=0.001), and poorer OS outcomes (42.3% vs. 90.7%, p=0.002). Multivariate analysis revealed that the HPV-associated cervical cancer maintained independent prognostic significance for both PFS (HR = 3.44, p=0.035) and OS (HR = 6.83, p=0.033) (50).

Regarding immunotherapy with aniti-PD-1/PD-L1 antibody, pretreatment serum CRP concentrations emerged as independent predictors of both PFS and OS rates in R/M cervical cancer patients undergoing immunotherapeutic interventions (53). PD-L1-expressing circulating tumor cells serve as prognostic biomarkers in patients with cervical cancer receiving chemoradiotherapy (54). Moreover, PD-1 blockade therapy substantially enhances radiation treatment effectiveness (55). The synergistic application of immunotherapeutic agents with palliative radiation was strongly associated with CR (odds ratio [OR] =6.31; p=0.005). Comparative analysis revealed superior 36-month PFS (73.7% vs. 33.8%, p=0.0048) and OS rates (85.7% vs. 38.7%, p=0.0043) in the combination therapy cohorts. Individuals who underwent more than four immunotherapy cycles demonstrated superior PFS (69.9% vs. 15.2%; p<0.001) and enhanced OS (64.6% vs. 39.7%; p=0.032). Patients presenting solely with recurrence exhibited prolonged survival relative to those with metastatic involvement or combined presentations (77.7% vs. 44.4% vs. 40.1%, respectively; p=0.024). Importantly, patients with squamous cell carcinoma achieved markedly higher 24-month PFS rates than those with other histological subtypes (57.9% vs. 14.6%; p=0.042) (56). The systemic immune-inflammation index (SII) demonstrates significant prognostic value as an independent predictor of PFS. This association, supported by clinical evidence, suggests that SII could serve as a reliable prognostic indicator for monitoring treatment responses in patients with cervical cancer undergoing immunotherapy (57). A meta-analysis of phase 3 randomized controlled trials has revealed that combining PD-1/PD-L1 inhibitors with chemotherapy demonstrated greater efficacy than chemotherapy monotherapy in treating advanced cervical cancer (R/M or locally advanced), enhancing both OS and objective response rates (58). Furthermore, a subsequent phase 2 clinical investigation involving patients with R/M cervical cancer who discontinued standard therapies owing to inefficacy or intolerance reported a median PFS of 7.16 months. The median OS remains undetermined in the initial analysis (59). A phase II multicenter trial employing a single-arm, open-label design evaluated Enlonstobart, an innovative PD-1 inhibitor, in patients with PD-L1-positive cervical cancer with disease progression or intolerance to initial platinum-based chemotherapy. This study obtained a DCR of 54.2%. Patients exhibited a median response duration of 16.6 months, and the PFS averaged 3.1 months across the cohort. The median OS endpoint remained unreached at the time of the data cutoff (60). Regarding immunotherapy with camrelizumab regimens, a multicenter phase II clinical trial (CLAP) with an open-label, single-arm design evaluated camrelizumab combined with apatinib in advanced cervical cancer cases. The data revealed that 57.8% of the participants had undergone two or more chemotherapy regimens for R/M conditions, whereas 22.2% had prior bevacizumab exposure. Clinical outcomes demonstrated an overall response rate (ORR) of 55.6% with a median PFS of 8.8 months (61). Subsequent analysis of the CLAP trial indicated a median duration of response of 16.6 months, with 45.0% of responders attaining sustained therapeutic effects lasting ≥24 months. The PFSl rate at 1 year was 40.7%, which decreased to 37.8% by 18 months. The median OS was 20.3 months, with a 24-month survival rate of 47.8%. Several prognostic indicators demonstrated significant correlations with enhanced PFS and extended OS, including patients aged >50 years, PD-L1 expression levels (CPS ≥ 1 compared to <1 and CPS ≥ 10 versus <1), elevated tumor mutational burden, and presence of PIK3CA genetic alterations (62). *Post-hoc* evaluation of the CLAP trial data demonstrated that mutational changes affecting PIK3CA, PTEN, ERBB3, and components of the PI3K/AKT signaling cascade, combined with tumor mutational burden, could represent innovative prognostic indicators in individuals receiving anti-PD-1-based combination treatments for cervical carcinoma (63). An open-label, phase 2 trial investigating the combination therapy of camrelizumab with nab-paclitaxel and carboplatin demonstrates promising efficacy and manageable toxicity profiles in patients with R/M cervical cancer (64).

Our analysis revealed several key factors associated with extended survival duration. The first was HPV status. The patient had HPV-associated cervical cancer. CR rates have been reported to reach 57.5% among the study participants, with the HPV-associated cohort exhibiting significantly higher CR rates than the HPV-independent cohort (81.8% vs. 27.8%, p=0.001) (50). Patients with cervical carcinoma who exhibited detectable levels of HPV-derived cell-free DNA either immediately following treatment completion or 3 months afterward demonstrated markedly worse prognostic indicators than HPV-negative cases (65). The second key factor was positive PD-L1. Incorporating immune checkpoint inhibitors into first-line standard-of-care regimens for recurrent or advanced cervical cancer enhanced clinical outcomes. This combined therapeutic efficacy may be most beneficial among specific patient cohorts, particularly in individuals with elevated PD-L1 expression levels (66). Immunotherapy targeting the PD-1/PD-L1 pathway showed enhanced clinical benefits for PD-L1-positive cervical cancer patients, manifesting improved ORR, DCR, median PFS, and median OS periods (67). The last key factor was stage IIIC2. Although trials have assessed outcomes in stage IVB, persistent, and recurrent cervical cancer, new treatments have demonstrated poorer PFS and OS for stage IVB than for persistent and recurrent cervical cancer (68).

This clinical scenario raises several noteworthy considerations concerning diagnostic approaches and therapeutic management strategies that merit careful examination. First, regarding postoperative radiotherapy, based on the Sedlis criteria, the patient exhibited moderate-risk characteristics, including deep cervical stromal invasion, a neoplasm measuring 2.5 cm in diameter, and a lack of lymphovascular involvement. The treatment plan included adjuvant radiotherapy as prescribed. However, after completing the fifth radiation therapy session, the patient opted to discontinue treatment prematurely because of personal considerations. Second, for first-line therapy, the patient received a combination of chemotherapy and radiation therapy, excluding subsequent administration of bevacizumab, immune checkpoint inhibitors, or combination immunotherapy. Recent advancements in primary cervical cancer management have demonstrated that incorporating pembrolizumab into platinum-containing chemotherapy regimens, whether administered alongside bevacizumab or as monotherapy, yields superior clinical results compared to conventional platinum-based chemotherapy combined solely with bevacizumab (69). A retrospective multicenter longitudinal analysis evaluated three distinct cohorts of patients with R/M cervical cancer. Cohort 1 received frontline chemotherapy supplemented with bevacizumab, immune checkpoint inhibitors, or combination therapy; cohort 2 received cytotoxic chemotherapy alone; and cohort 3 received no antineoplastic interventions. Survival analysis revealed significant differences, with cohort 1 demonstrating a median PFS duration of 12 months versus 8.8 months in cohort 2 and 3 months in cohort 3. The corresponding median OS measurements were 25, 17, and 3.6 months in the treatment groups, respectively (70). Lastly, regarding anti-vascular endothelial growth factor targeted therapy, the patient did not receive bevacizumab during the first- or second-line treatment. The administration of this agent may have contributed to OS improvement. Clinical evidence indicates that bevacizumab demonstrates enhanced OS outcomes when integrated into chemotherapeutic regimens compared to chemotherapy as monotherapy in patients with cervical cancer (8). A comprehensive meta-analysis evaluating bevacizumab combined with chemotherapy for cervical cancer treatment revealed clinically meaningful enhancements in PFS and OS outcomes (71).

Our case report illustrates that second-line therapy utilizing camrelizumab in combination with nab-paclitaxel exhibits notable efficacy and manageable safety profile. However, multi-center, randomized phase II/III clinical trials are warranted to explore whether this regimen can be clinically applied to a broader population of patients with R/M cervical cancer as a second-line treatment.

## Data Availability

The raw data supporting the conclusions of this article will be made available by the authors, without undue reservation.
